# Inpatient antibiotic utilization in the Veterans’ Health Administration during the coronavirus disease 2019 (COVID-19) pandemic

**DOI:** 10.1017/ice.2020.1277

**Published:** 2020-10-20

**Authors:** Thomas D. Dieringer, Daisuke Furukawa, Christopher J. Graber, Vanessa W. Stevens, Makoto M. Jones, Michael A. Rubin, Matthew Bidwell Goetz

**Affiliations:** 1 David Geffen School of Medicine at UCLA, Los Angeles, California; 2 Veterans’ Affairs Greater Los Angeles Healthcare System, Los Angeles, California; 3 Veterans’ Affairs Salt Lake Informatics, Decision-Enhancement, and Analytic Sciences (IDEAS) Center, VA Salt Lake City Health Care System, Salt Lake City, Utah; 4 University of Utah School of Medicine, Salt Lake City, Utah

## Abstract

Antibiotic prescribing practices across the Veterans’ Health Administration (VA) experienced significant shifts during the coronavirus disease 2019 (COVID-19) pandemic. From 2015 to 2019, antibiotic use between January and May decreased from 638 to 602 days of therapy (DOT) per 1,000 days present (DP), while the corresponding months in 2020 saw antibiotic utilization rise to 628 DOT per 1,000 DP.

Inpatient antimicrobial use has decreased in the Veterans’ Health Administration (VA) over the past decade.^1^^,^^2^ However, with the onset of the coronavirus disease 2019 (COVID-19) pandemic, the presence of, or concern for, bacterial coinfection and the development of secondary infections in confirmed and suspected COVID-19 disease are potential drivers of increased antimicrobial use. Reports of increased antibiotic use during the COVID-19 pandemic have generally been from heavily impacted facilities or geographic regions and provide few details regarding patterns of antibiotic use.^1–3^ To provide a broader overview of changes in antibiotic use in a healthcare system with a long-standing enterprise-wide commitment to antibiotic stewardship, we compared patterns of antibiotic use throughout the VA health system from January through May 2020 with corresponding periods in prior years.

## Methods

Data on antibacterial use from 2015 through 2020 were extracted from the VA Corporate Data Warehouse for acute inpatient care units in all VA facilities excluding those that provide limited acute inpatient services using previously described methods.^2^ To reduce the impact of seasonal effects, only data from January 1 to May 31 for each year were included. Days of therapy (DOT) per 1, 000 days present (DP) were calculated and stratified by CDC-defined standardized antimicrobial administration ratio (SAAR) antibiotic classes.^4^

Because of changes in healthcare utilization that have accompanied the COVID-19 pandemic, we also examined changes in the total number of days of antibiotic therapy to provide insight into whether more days of therapy were being administered or whether the changes in therapy reflected the same amount of antibiotic use distributed across a smaller hospital population. To evaluate the impact of initial COVID-19 case burden, we conducted a subset analysis excluding facilities with the highest initial COVID-19 case loads (New England, New York, New Jersey, Michigan, Chicago, and New Orleans). This study was approved by the VA Central Institutional Review Board.

## Results

Data were available for 84 inpatient VA facilities. From 2015 through 2019, antibiotic use during January–May of each year decreased from 638 to 602 DOT per 1,000 DP (mean decrease, 9.1 DOT per 1,000 DP per year) (Table 1A). Consistent year-to-year decreases were observed for broad-spectrum agents used for hospital-onset infections (−2.4 DOT per 1,000 DP), broad-spectrum agents used for community-onset infections (−5.2 DOT per 1,000 DP), and agents used for resistant gram-positive infections (−5.1 DOT per 1,000 DP). Consistent increases occurred in the use of narrow-spectrum β-lactam agents (2.9 DOT per 1,000 DP). The same reversal in the trends (up and down) in antibiotic use were observed when facilities in regions with the highest initial rates of COVID-19 were excluded (Table 1).

**Table 1.** Trends in Antibiotic Use by CDC Drug Class, DOT/1,000 Days Present

Year	Narrow ß-lactams	Broad Community^a^	Broad Hospital^a^	Anti-MDRO^a^	Anti-MRSA^a^	All Other	Total
	**DOT per 1,000 days present, all eligible facilities**						
2015	82	141	155	2.0	122	137	638
2016	84	136	152	1.5	117	136	627
2017	87	130	150	1.7	113	135	617
2018	92	127	146	1.8	107	136	609
2019	93	120	145	1.7	101	141	602
**Change/year**	**2.9**	**−5.2**	**−2.4**	**−0.1**	**−5.1**	**0.9**	**−9.1**
2020	89	129	152	1.7	103	153	628
**2020 vs 2019**	**−4.5**	**9.1**	**7.5**	**0.1**	**1.6**	**12.2**	**25.9**
	**DOT per 1,000 days present, excluding facilities heavily impacted by COVID-19^b^**						
2015	82	148	158	2.2	126	140	656
2016	83	141	154	1.6	120	137	637
2017	86	134	153	1.8	117	136	628
2018	92	132	148	1.9	109	138	621
2019	94	123	146	1.7	104	142	611
**Change/year**	**3.1**	**−6.1**	**−3.1**	**−0.1**	**−5.6**	**0.5**	**−11.3**
2020	91	132	154	1.7	106	154	638
**2020 vs 2019**	**−3.4**	**8.4**	**8.2**	**0.0**	**2.1**	**12.0**	**27.3**

Note. CDC, Centers for Disease Control and Prevention; MDRO, multidrug-resistant organism; MRSA, methicillin-resistant *Staphylococcus areus*; DOT, days of therapy.

a Broad community, broad hospital, anti-MDRO and anti-MRSA, respectively, refer to agents used for community-onset infections, agents used for hospital-onset infections, agents used for MDROs and antibacterial agents predominantly used for resistant gram-positive infections (eg, MRSA).

b Facilities in New England, New York, New Jersey, Michigan, Chicago, and New Orleans are excluded.

In contrast, antibiotic use in the same period in 2020 increased from 602 to 628 DOT per 1,000 DP. Increases were most prominent for agents not within one of the Centers for Disease Control and Prevention (CDC)-defined SAAR classes (12.2 DOT per 1,000 DP), broad-spectrum agents used for community-onset infections (9.1 DOT per 1,000 DP), and broad-spectrum agents used for hospital-onset infections (7.5 DOT per 1,000 DP). Use of narrow-spectrum β-lactam agents decreased (−4.5 DOT per 1,000 DP). Lesser changes were observed in the use of antibacterial agents predominantly used for resistant gram-positive infections (1.6 DOT per 1,000 DP). The greatest increases in the use of individual antibiotics (DOT per 1,000 DP) were for ceftriaxone (14.7 DOT per 1,000 DP), cefepime (10.5 DOT per 1,000 DP), doxycycline (6.2 DOT per 1,000 DP), and azithromycin (6.2 DOT per 1,000 DP).

For the period of January–May in 2015 through 2019, the number of acute–care DP at all VA facilities was 1,245,309 ± 31,178 (mean ± SD), and the total antibiotic DOT were 770,799 ± 35,288 (Table 2). In 2020, DP decreased to 1,024,473, representing a decrease of 174,182 DP from 2019 (14.5% decrease), and total antibiotic DOT decreased to 643,455, a decrease of 78,306 from 2019 (10.8% decrease). Compared with 2019, DP and DOT decreased starting in March reaching a nadir of −32% and −23% for April 2020.

**Table 2.** Days Present and Antibiotic Days of Therapy (DOT)^a^

Year	Antibiotic DOT							
	Days Present	Narrow ß-lactams	Broad Community	Broad Hospital	Anti-MDRO	Anti-MRSA	All Other	Total
2015	1,291	106	182	200	2.6	157	177	824
2016	1,265	106	173	192	1.9	148	172	793
2017	1,242	109	161	187	2.1	140	167	766
2018	1,230	113	156	179	2.2	131	168	749
2019	1,199	112	144	174	2.0	121	169	722
**Change per year**	**−23**	**1.5**	**−9.5**	**−6.5**	**−0.1**	**−9.0**	**−2.0**	**−26**
2020	1024	91	132	156	1.8	105	171	643
**2020 vs 2019**	**−174**	**−21**	**−12**	**−18**	**−0.2**	**−16**	**−12**	**−78**

Note. MDRO, multidrug-resistant organism; MRSA, methicillin-resistant *Staphylococcus areus*.

a Days are in thousands.

## Discussion

We have demonstrated a substantial increase in the density of antimicrobial utilization during the period of January–May of 2020 at 84 VA medical facilities, which largely negated the downward trend of antibiotic use achieved through antimicrobial stewardship efforts over the prior 5 years.^5^ The largest increase in the rate of use was for antibiotics that are typically used for empiric therapy for community-acquired pneumonia (CAP), but increases were also seen in the use of broad-spectrum antibiotics that are typically used to treat hospital-acquired pathogens.

In addition to considering the density of antibiotic use (DOT per 1,000 DP), we also evaluated total antibiotic use to partially account for decreases in healthcare utilization both for elective procedures and emergency conditions during the COVID-19 pandemic. The absolute number of antibiotic days decreased, albeit at a lesser rate than did the number of hospital days. However, although overall institutional use of antibiotics has decreased, the increased density of antibiotic use as measured by the use per patient day may still adversely impact patient-level outcomes and institutional antimicrobial resistance patterns.

Our findings confirm and extend prior smaller-scale studies. A single-center study at an academic hospital in Virginia reported significantly increased use of ceftriaxone and azithromycin but not of other broad-spectrum antibiotics coincident with the onset of the pandemic.^1^ Another single-center study from Spain showed increased use of amoxicillin-clavulanate during the early phase of the pandemic, followed by later increased utilization of broad-spectrum antibiotics.^2^

There are several potential explanations for the observed increases in antimicrobial use including concerns of bacterial coinfection in suspected or newly diagnosed COVID-19 patients, increased risk of nosocomial infection due to administration of immunomodulatory therapy, reluctance to obtain diagnostic respiratory specimens,^3^ diversion of clinical resources from stewardship activities during a time of crisis, and an increased proportion of hospitalizations due to respiratory infections, which typically prompt antibiotic therapy. Our finding that increases in antibiotic use were generalized across the VA and not restricted to facilities in areas with the highest case burden of COVID-19 suggests that issues not directly related to the care of COVID-19 patients contribute to the increased density of antibiotic use.

The World Health Organization recommends prompt administration of empiric antimicrobials in persons with suspected or confirmed severe COVID-19.^6^ In view of the similarities in the presentation of bacterial CAP and severe COVID-19, the co-chairs of the 2019 American Thoracic Society and Infectious Diseases Society of America CAP guidelines recommend empiric antibiotics for patients with CAP without confirmed COVID-19 while indicating that antibiotics are not required in all patients with confirmed COVID-19–related pneumonia.^7^

However, the value of routine antibiotic therapy for patients with confirmed COVID-19 is questionable. While including publications with differing definitions of infection, and inconsistent timing and settings of sample collection, it is notable that recent meta-analyses found that the estimated rates of identified initial co-infection in COVID-19 cases average 3.5% while reports of secondary bacterial infection emerging during the course of hospitalization ranged from 0% to 45.5% with pooled rates of 7%–8%.^8^^–^^10^

This study has several limitations. First, although this was a multicenter study, the participating institutions were all VA facilities, which may limit generalizability. Additionally, we did not analyze patient-level data; thus, appropriateness, indication, or duration of antibiotics were not evaluated. We did not assess the degree to which each facility was affected by the pandemic, changes in the composition of the hospitalized patient populations, or other facility characteristics that may influence antimicrobial use. Future studies looking specifically at appropriateness of antibiotics administered to patients with COVID-19 should examine these issues.
